# A Meta-Analysis on the Effect and Safety of LCZ696 in the Treatment of Hypertension

**DOI:** 10.1155/2021/8867578

**Published:** 2021-03-20

**Authors:** Li Zheng, Binbin Xia, Xuqian Zhang, Yan Zhao

**Affiliations:** ^1^Department of Pharmacy, China Aerospace Science & Industry Corporation 731 Hospital, Beijing 100074, China; ^2^Department of Pharmacy, Luhe Hospital, Beijing 101149, China; ^3^Department of Gastroenterology and Hepatology, China Aerospace Science & Industry Corporation 731 Hospital, Beijing 100074, China

## Abstract

**Objectives:**

To systematically evaluate the differences in effect and safety of LCZ696 and angiotensin receptor blockers (ARBs) in the treatment of hypertension.

**Methods:**

We searched PubMed, Cochrane, Web of Science, and Ovid, collected randomized controlled trials (RCTs) about the effect and safety of LCZ696 and ARBs in hypertensive patients, extracted relevant data and evaluated the quality of the included literature according to the RCT quality evaluation standard recommended by Cochrane Reviewer's Handbook, using RevMan 5.3, and performed meta-analysis.

**Results:**

Eight RCTs studies were included, with a total of 4313 patients. Compared with ARBs, LCZ696 can better reduce systolic blood pressure (msSBP) (WMD −4.29 mmHg; 95% CI: −5.37 to −3.21; *P* < 0.001), diastolic blood pressure (msDBP) (WMD −1.87 mmHg; 95% CI:−2.38 to −1.36; *P* < 0.01), ambulatory systolic blood pressure (maSBP) (WMD −3.37 mmHg; 95% CI:−4.26 to −2.47; *P* < 0.01), and ambulatory diastolic blood pressure (maDBP) (WMD −1.47 mmHg; 95% CI: −1.97 to −0.97; *P* < 0.01). In terms of safety, LCZ696 is basically the same as ARBs, but LCZ696 is more likely to cause cough than ARBs (OR = 2.38; 95% CI: 1.27 to 4.47; *P* < 0.01).

**Conclusion:**

LCZ696 can significantly reduce the blood pressure level of patients with hypertension, but it is necessary to pay attention to whether the patient will experience coughing after taking the drug.

## 1. Introduction

Hypertension is a chronic condition that contributes to worldwide morbidity and mortality more than any other risk factor. As reported, the number of patients with hypertension was estimated to be 31.1% among adults in 2010 [1]. Hypertension is the most common cardiovascular leading risk factor and underlies heart failure (HF), coronary artery disease, stroke, and chronic kidney disease. Hypertensive heart disease includes the development of diastolic dysfunction, left ventricular hypertrophy, and HF with preserved and reduced ejection fraction [2]. Therefore, the optimal treatment of hypertension can prevent the development of HF.

Blood pressure can be controlled by changing diet and lifestyle, but drug treatment is used when these methods are not effective. Commonly prescribed antihypertensive drugs include thiazide-diuretics, *β*-blockers, calcium channel blockers, angiotensin converting enzyme (ACE) inhibitors, and angiotensin II receptor blockers (ARB) [3]. However, lots of treated patients still cannot effectively control blood pressure level, and we need to keep looking for better antihypertensive agents to achieve BP goals and reduce cardiovascular events.

Various studies have revealed the potential of LCZ696 as an antihypertensive drug [4–6]. LCZ696 provides highly selective inhibition of neprilysin and blockade of the angiotensin receptor. It plays an important role in improving HF, reducing ejection fraction, and controlling blood pressure [7, 8]. Here, we searched studies and performed a meta-analysis on available randomized clinical (RCT) trials to research the effect and safety of LCZ696 in hypertension patients.

## 2. Methods

**Figure 1:**
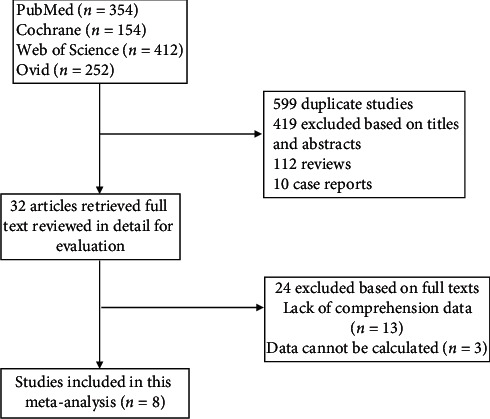
PRISMA flowchart.

Our search strategy is illustrated in the Preferred Reporting Items for Systematic Reviews and Meta-analyses (PRISMA) [9] reporting guideline flowchart in Figure 1.

### 2.1. Search Strategy

All data were obtained by actively searching PubMed, Cochrane, Web of Science, and Ovid up to Sep 1, 2020. Keywords and Medical Subject Headings (MeSH) included the following: “LCZ696,” “sacubitril/valsartan,” “AHU377,” “angiotensin receptor-neprilysin inhibitor (ARNi),” and “hypertension.”

### 2.2. Inclusion Criteria

Studies meeting the following criteria were selected:

1. The trial was a RCT with complete data
2. Patients with clinical diagnosis of hypertension
3. LCZ696 was used in the experimental group (any dose)
4. ARBs were used in the control group
5. There were adverse events (AEs) in the outcome indicators

### 2.3. Exclusion Criteria

Studies that used only self-report or informant report as an outcome were excluded. We excluded animal experimental studies as well as case reports from the meta-analysis. In the same way, all studies that are not written in English, non-RCT studies, duplicate studies, single-arm studies, review articles, and conference articles were eliminated.

### 2.4. Extraction of Data

Study data were extracted by two of the authors. Studies that clearly did not meet the inclusion criteria were excluded by reading the title and abstract. After reading the full text, they decided whether to include these studies which may meet the inclusion criteria. If there was a problem of disagreement between the two authors, it was resolved through negotiation or decided after discussion with the third researcher.

### 2.5. Study Quality

The risk of bias of RCTs was assessed using the Cochrane Collaboration's tool from 7 aspects:

1. Random sequence generation (selection bias)
2. Allocation concealment (selection bias)
3. Blinding of participants and personnel (performance bias)
4. Blinding of outcome assessment (detection bias)
5. Incomplete outcome data (attrition bias)
6. Selective reporting (reporting bias)
7. Other bias

### 2.6. Outcome Indicators

In eligible articles, the main indicators extracted were the mean reductions in systolic blood pressure and diastolic blood pressure in the sitting position (msSBP, msDBP), the mean reductions in 24-hour ambulatory SBP (maSBP) and 24-hour ambulatory DBP (maDBP), and the number of participants who achieved BP, msSBP, and msDBP control. The safety outcome was any AEs occurring during the follow-up point.

### 2.7. Statistical Analysis

This meta-analysis was performed using the statistical software RevMan 5.3. For the continuous variables, such as msSBP, msDBP, maSBP, and maDBP, the data was represented as the weighted mean difference (WMD) between the experimental group and the control group, with a 95% confidence interval (CI), and we performed subgroup analysis to evaluate the effect about BP control based on different doses of LCZ696. For binary variables, such as AEs, the effect size was represented as an odds ratio (OR) with a 95% CI. The chi-square test was used to assess heterogeneity between studies. When *P* > 0.1 or *I*^2^<50%, the fixed-effect model was applied; otherwise, the random-effect model was used for data analysis.

### 2.8. Publication Bias

Funnel plots and Egger test were used to assess potential publication bias. When two-sided *P* value < 0.05, it means there was publication bias.

### 2.9. Sensitivity Analysis

We conducted sensitivity analysis. In the RevMan5.3 software, the included studies were removed one by one, and the stability of the results was evaluated by analyzing the changes in the pooled WMD and the pooled OR.

## 3. Results

### 3.1. Study Characteristics

1172 relevant studies had been found from the above-mentioned databases. According to the inclusion and exclusion criteria, 8 studies [10–17] were finally included. There were 4313 patients, 1917 patients were randomly assigned to the ARBs group, and 2396 patients were randomly assigned to the LCZ696 group.

Studies population included untreated patients (newly diagnosed patients or those who had not been taking any antihypertensive drug for ≥4 weeks before screening) or treated with antihypertensive drugs for ≥4 weeks before screening, and mean sitting systolic BP (SBP) ≥140 to <180 mm Hg at screening (for untreated patients only) or ≥120 to ≤160 mm Hg at screening and ≤180 mm Hg at the end of the washout period (for treated patients only). Exclusion criteria were patients with severe hypertension, history of angioedema, and previous or current diagnosis of HF (NYHA class II-IV).

The 8 included studies were all clinical RCTs, of which 5 studies compared LCZ696 with olmesartan, and the remaining 3 studies compared LCZ696 with valsartan. The age of patients ranged from 50 to 75 years. Male patients were the majority. The treatment period ranged from 4 weeks to 52 weeks. Some basic characteristics of the included studies are collected in Table 1.

### 3.2. Study Quality

**Figure 2:**
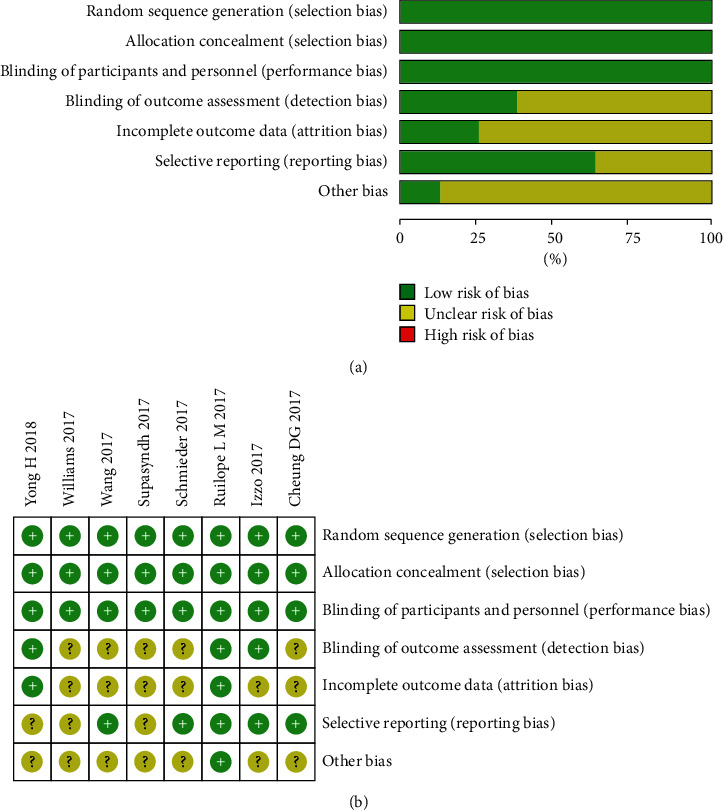
Risk assessment of bias in clinical studies.

Quality of studies was assessed by the Cochrane Collaboration's Risk of Bias tool and all included have acceptable quality. All trials were multicenter, randomized, double-blind, active-controlled, parallel-group studies. The included studies were all high-quality articles (level A). Detailed information on the risk of bias is shown in Figure 2.

### 3.3. Analysis of Blood Pressure Control Results

We mainly included 5 outcome indicators to compare the effect of the drug LCZ696 and ARBs, including msSBP, msDBP, maSBP, maDBP, and blood pressure control rate.

**Figure 3:**
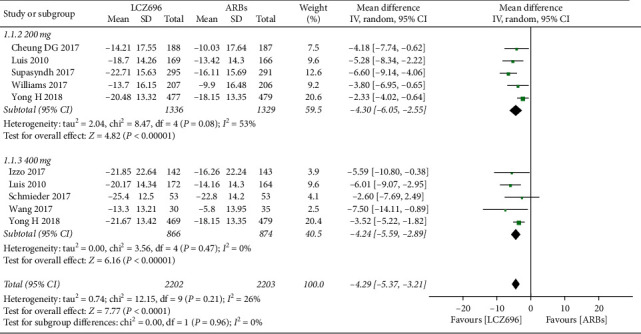
Comparison of LCZ696 with ARBs on mean reduction (mm Hg) in sitting systolic blood pressure (msSBP).

**Figure 4:**
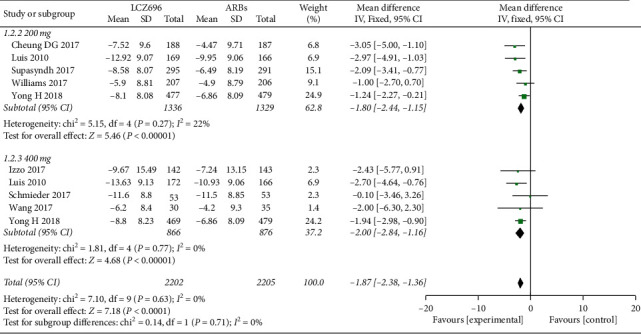
Comparison of LCZ696 with ARBs on mean reduction (mm Hg) in sitting diastolic blood pressure (msDBP).

**Figure 5:**
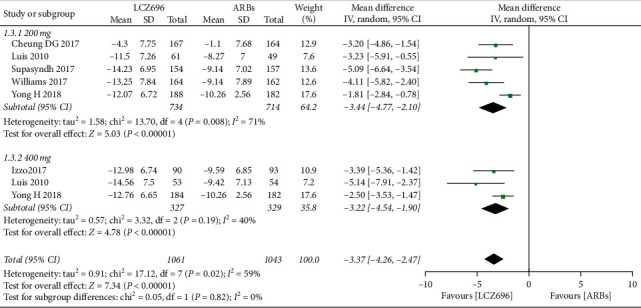
Comparison of LCZ696 with ARBs on mean reduction (mm Hg) in ambulatory systolic blood pressure (maSBP).

**Figure 6:**
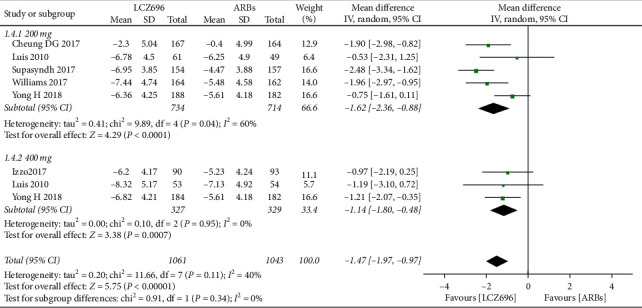
Comparison of LCZ696 with ARBs on mean reduction (mm Hg) in ambulatory diastolic blood pressure (maSBP).

The pooled WMD results showed that LCZ696 had a more pronounced antihypertensive effect compared with the results of ARBs treatment. Indeed, LCZ696 can significantly reduce msSBP (WMD −4.29 mmHg; 95% CI: −5.37 to −3.21; *P* < 0.001; *I*^2^ = 26%) (Figure 3), msDBP (WMD −1.87 mmHg; 95% CI:−2.38 to −1.36; *P* < 0.001; *I*^2^ = 0) (Figure 4), maSBP (WMD −3.37 mmHg; 95% CI:−4.26 to −2.47; *P* < 0.001; *I*^2^ = 59%) (Figure 5), and maDBP (WMD −1.47 mmHg; 95% CI:−1.97 to −0.97; *P* < 0.001; *I*^2^ = 40%) (Figure 6).

According to the different doses of LCZ696, we did a subgroup analysis. Taking 200 mg daily of LCZ696 treatment showed a more significant reduction in msSBP (WMD −4.30 mmHg; 95% CI:−6.05 to −2.55; *P* < 0.001; *I*^2^ = 53%), msDBP (WMD −1.80 mmHg; 95% CI:−2.44 to −1.15; *P* < 0.001; *I*^2^ = 22%), maSBP (WMD −3.44 mmHg; 95% CI:−4.77 to −2.10; *P* < 0.001; *I*^2^ = 71%), and maDBP (WMD −1.62 mmHg; 95% CI:−2.36 to −0.88; *P* < 0.001; *I*^2^ = 60%). Taking 400 mg daily of LCZ696 treatment also showed a more significant reduction in msSBP (WMD −4.24 mmHg; 95% CI:−5.59 to −2.89; *P* < 0.001; *I*^2^ = 0), msDBP (WMD −2.00 mmHg; 95% CI:−2.84 to −1.16; *P* < 0.001; *I*^2^ = 0), maSBP (WMD −3.22 mmHg; 95% CI:−4.54 to −1.90; *P* < 0.001; *I*^2^ = 40%), and maDBP (WMD −1.14 mmHg; 95% CI:−1.80 to −0.48; *P* < 0.001; *I*^2^ = 0)

**Figure 7:**
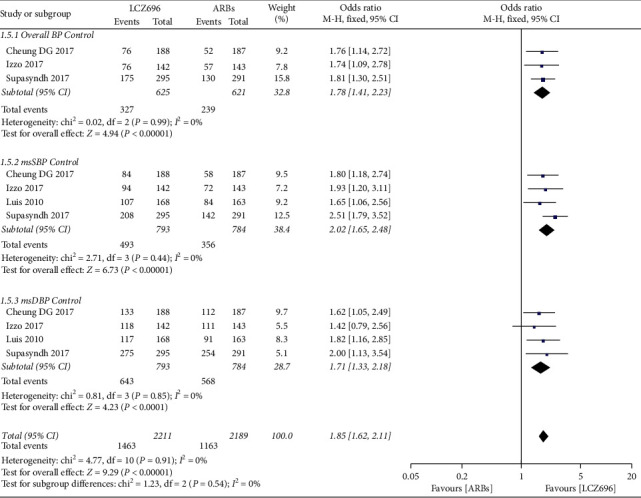
Comparison of LCZ696 with ARBs on BP control.

In addition, LCZ696 had more advantages than ARBs in the control of overall BP (OR = 1.78; 95% CI:1.41 to 2.23; *P* < 0.001; *I*^2^ = 0), msSBP (OR = 2.02; 95% CI:1.65 to 2.48; *P* < 0.001; *I*^2^ = 0), and msDBP (OR = 1.71; 95% CI:1.33 to 2.18; *P* < 0.001; *I*^2^ = 0) (Figure 7).

### 3.4. AEs

All included studies had reported several drug-related adverse events after treatment with LCZ696 or ARBs [10–17]. A pooled analysis of all adverse events data showed that there were statistical differences between LCZ696 and ARBs groups in adverse events (OR = 1.13; 95% CI:1.02 to 1.24; *P* < 0.05; *I*^2^ = 0). The results were more favorable to the ARBs group. More exactly, in all the included studies, LCZ696 could increase the incidence of cough in patients (OR = 1.71; 95% CI:1.33 to 2.18; *P* < 0.001; *I*^2^ = 0). But except for cough, the results of every adverse event that occurred showed no statistical difference between the LCZ696 and ARBs groups. The detailed analysis of the data is shown in Table 2.

### 3.5. Publication Bias Review

This meta-analysis only included 8 studies. According to the requirements of the Cochrane Reviewer's Handbook, the funnel chart generally requires more than 10 studies to be meaningful; otherwise, its test power will decrease, and the evaluation of publication bias is not accurate, so publication bias evaluation was not performed.

### 3.6. Sensitivity

Among the 8 studies included in this meta-analysis, different types of studies were eliminated in turn through the analysis of the study characteristics and the differences in patient baseline characteristics; the remaining studies were combined with statistics (pooled WMD or pooled OR) to perform the meta-analysis again. There was no difference between the merged result and the original result.

## 4. Discussion

LCZ696 combines the actions of an ARB and valsartan with a neprilysin inhibitor sacubitril [7]. It is the first new drug known as a dual inhibitory effect.

The main factors and pathogenesis of hypertension are generally believed to be due to the fluid retention and renin-angiotensin-aldosterone system (RAAS) excessive activation. Other studies also had shown that the excessive activation of the sympathetic nervous system (SNS) promotes the reabsorption of the kidneys and damages the pressure natriuretic effect, thereby inducing hypertension [18]. The activation of the RAAS system is caused by many reasons. Angiotensin II promotes smooth muscle contraction, increases ventricular cell synthesis, aldosterone secretion, and arterial smooth muscle cell growth, and can also increase blood pressure. Valsartan inhibits the abnormal activation of the RAAS system by inhibiting angiotensin II receptors, thereby reducing peripheral vascular resistance and reducing fluid retention by inhibiting vasoconstriction and aldosterone secretion. Neprilysin is a neutral endopeptidase that degrades several potentially beneficial vasoactive peptides, including natriuretic peptides, bradykinin, and adrenomedullin, and sacubitril can enhance the effects of these peptides by inhibiting neprilysin, thus promoting vasodilation and increasing urine volume [10]. Therefore, we believe that neprilysin inhibition simultaneously with angiotensin receptor blockade had the potential to counteract some of the mechanisms contributing to arterial stiffening in patients with systolic hypertension and could lower central aortic pressure more effectively than an ARB alone.

An experiment called PARADIGM HF studied by JV McMurray et al. included 8442 patients with HF (NYHA II—IV, LVEF≤40%, 88% ejection fraction less than 35%). The therapeutic effect of enalapril on patients with HF was compared with LCZ696. The results showed that LCZ696 can significantly reduce the cardiovascular mortality and HF-related hospitalization rates in patients, and it is better than enalapril in clinical symptoms and long-term prognosis for patients with HF [19]. In addition, LCZ696 can prevent cardiovascular disease and renal insufficiency and has antichronic kidney disease (CKD) and anticardiovascular disease (CVD) effects [20, 21]. For patients with severe HF, it is necessary to distinguish between advanced HF (AdHF) and end-stage HF. AdHF patients have intolerance to drugs acting on the renal axis, i.e., ACE-I, ARB, or LCZ696, due to hypotension and/or worsening renal function, with risk of 20% for mortality in 1 year. But end-stage HF patients have an extremely severe cardiomyocyte dysfunction and irreversible organ dysfunction and do not respond to inotropic drugs. The presence of the aforementioned features suggests an irreversible condition of end-stage HF, for which drug treatment or advanced therapies, such as ventricular-assist device implantation, are contraindicated and palliative cares should be pursued [22].

Hypertension is a reversible factor of early HF. Long-term hypertension will increase the ejection resistance of the heart and thicken the ventricular wall, which will lead to ventricular remodeling and chronic HF [23]. Hypertension is the most important risk factor for death from cardiovascular and cerebrovascular diseases, bringing a heavy burden of disease to society. In 2017, the number of deaths due to hypertension in China reached 2.54 million, of which about 69% were deaths from stroke, 54% were deaths from ischemic heart disease, 41% were deaths from other cardiovascular diseases, and another 43% were deaths from chronic kidney disease due to high blood pressure [24]. There are also studies showing that hypertension is a high risk factor for Alzheimer's disease [25]. As mentioned in Severino P. et al.'s article [26], risk factors, such as hypertension, diabetes, hyperlipidemia, smoking, and inflammation, not only can cause the pathological changes of large blood vessels, but also are pathogenic factors of coronary microvessels. These risk factors can lead to abnormal microvascular function through endothelial cell-dependent and independent mechanisms and eventually lead to ischemic heart disease and heart failure. LCZ696 can effectively lower blood pressure. Therefore, it can improve heart failure caused by microcirculation dysfunction, which is of great significance to patients with abnormal microcirculation function.

The efficacy test results show that LCZ696 has a better antihypertensive effect than the ARB drug valsartan alone [27], mainly because sacubitril and valsartan have a certain synergistic effect; on one hand, it inhibits the RAAS system; on the other hand, it can reduce fluid retention. From the perspective of safety, compared with ARBs, LCZ696 in this study showed no significant difference in safety except for a mild cough (these studies did not report patients who gave up LCZ696 treatment because they could not tolerate cough); that is, compared with ARB drugs alone, LCZ696 has the characteristics of a more significant antihypertensive effect and no obvious safety difference. Based on its remarkable effect and safety, LCZ696 may become one of the first-line drugs for the treatment of hypertension, but large sample experiments are still needed to confirm this conclusion.

This study systematically evaluated all currently included studies on the effect and safety of LCZ696 in blood pressure control. The research results showed that, compared with ARBs, LCZ696 can effectively reduce msSBP, msDBP, maSBP, and maDBP and improve blood pressure control rate. At the same time, compared with ARBs, LCZ696 only increased the incidence of cough, and there were no more adverse events when the dose of LCZ696 was increased. This showed that LCZ696 can benefit more hypertensive patients.

## 5. Limitations

First of all, this study strictly followed the inclusion and exclusion criteria and finally only included 8 studies. The number of studies was relatively small, and a larger research sample size is needed to fully reflect the effectiveness and safety of the drug. Secondly, the length of the included studies was inconsistent, and it lacked sufficient basis for the safety evaluation of long-term using LCZ696 in the treatment of hypertension.

## 6. Conclusion

Although this meta-analysis only included 8 studies and 4313 patients, the results showed that, compared with ARBs, LCZ696 showed better advantages in lowering blood pressure and can be used as an alternative antihypertensive drug.

## Figures and Tables

**Table 1:** Baseline characteristics of studies incorporated in the meta-analysis.

First author	Research design	*E*/*C*	Men, *m* (%)	Age (y)	BMI (kg/m^2^)	Baseline SBP (mmHg)	Baseline DBP (mmHg)	Duration	Number
Williams et al. [10]	Multicenter, randomized, double-blind, active-controlled, parallel-group study	LCZ696 200 mg, qd	52	68.2 ± 5.73	28.6 ± 4.47	160.4 ± 12.32	85.8 ± 8.62	52 weeks	229
		Olmesartan 20 mg, qd	52.4	67.2 ± 5.97	29.1 ± 4.9	160.8 ± 15.6	85.8 ± 8.6		225

Izzo et al. [11]	Multicenter, randomized, double-blind, placebo- and active-controlled, parallel-group study	LCZ696 400 mg, qd	50	61.2 ± 10.6	29.3 ± 5.5	159.6 ± 7.0	90.9 ± 8.9	8 weeks	142
		Valsartan 320 mg, qd	58	62 ± 11.5	30 ± 5.3	160.0 ± 7.3	90.2 ± 9.4		143
Wang et al. [12]	Multicenter, randomized, double-blind, crossover study	LCZ696 400 mg qd	64	55.7 ± 12.5	26.4 ± 3.8	147 ± 9.7	147.5 ± 12.1	4 weeks	36
		Valsartan 320 mg, qd	64	58.9 ± 7.5	25.7 ± 2.9	90.2 ± 6.9	90.4 ± 7.2		36

Supasyndh et al. [13]	Multicenter, randomized, double-blind, active-controlled, parallel-group study	LCZ696 200 mg, qd	48	70.5 ± 4.67	24.3 ± 3.15	160.5 ± 8.41	84.6 ± 9.74	14 weeks	296
		Olmesartan 20 mg, qd	52.1	70.9 ± 4.67	24.6 ± 3.24	160.0 ± 7.99	85.2 ± 9.83		292
Schmieder et al. [14]	Multicenter randomized, double-blind, double-dummy, active-controlled, parallel group study	LCZ696 400 mg, qd	64.9	60.5 ± 7.8	28.1 ± 4.5	155.3 ± 9.0	92.7 ± 8.8	52 weeks	57
		Olmesartan 40 mg, qd	70.2	59.2 ± 13.1	28.6 ± 3.9	155.0 ± 9.1	91.7 ± 8.7		57
Huo et al. [15]	Multicenter, randomized, double-blind, active-controlled, parallel-group study	LCZ696 200 mg, qd	52.6	57.5 ± 10.17	26.4 ± 3.91	158.0 ± 7.15	90.7 ± 9.37	8 weeks	479
		LCZ696 400 mg, qd	51.5	58.1 ± 9.71	26.3 ± 3.56	157.9 ± 6.73	89.8 ± 9.46		472
		Olmesartan 20 mg, qd	53.9	57.4 ± 10.14	26.4 ± 3.92	158 ± 6.53	90.8 ± 9.57		484

Cheung et al. [16]	Multicenter, randomized, double-blind, active-controlled, parallel-group study	LCZ696 200 mg, qd	51.6	57.1 ± 10.19	30.5 ± 5.86	157.1 ± 9.54	90.4 ± 10.24	8 weeks	188
		Olmesartan 20 mg, qd	50.8	58.0 ± 9.09	30.6 ± 5.09	157.8 ± 10.17	912 ± 8.89		187
Ruilope et al. [17]	Multicenter, randomized, double-blind, placebo- and active-controlled, parallel group, dose range study	LCZ696 100 mg, qd	61	53 ± 10.4	N/A	154.9 ± 11.89	99.9 ± 3.62	8 weeks	156
		LCZ696 200 mg, qd	54	54 ± 9.7		156.8 ± 11.98	99.9 ± 4.06		169
		LCZ696 400 mg, qd	56	52 ± 10.9		156.3 ± 12.32	100.4 ± 4.06		172
		Valsartan 80 mg qd	58	53 ± 9.6	N/A	154.8 ± 10.53	99.5 ± 4.10		163
		Valsartan 160 mg qd	59	53 ± 9.7		155.3 ± 10.79	99.8 ± 4.41		166
		Valsartan 320 mg qd	60	53 ± 10.1		156.0 ± 11.48	99.5 ± 3.63		164

**Table 2:** Adverse events (AEs) reported in the incorporated studies.

AEs	No. of studies (*n*)	LCZ696 group, *n*/*n*	ARBs group, *n*/*n*	Heterogeneity	Analysis model	OR	95% CI	*P*
All AEs	8	802/2396	612/1917	*P* = 0.5, *I*^2^ = 0	Fixed-effect model	1.13	0.99, 1.29	0.08
Discontinuations because of AEs	4	29/1865	24/1389	*P* = 0.58, *I*^2^ = 0	Fixed-effect model	1.09	0.62, 1.90	0.77
Serious AEs	5	37/1721	28/1245	*P* = 0.38, *I*^2^ = 5%	Fixed-effect model	1.14	0.69, 1.88	0.6
Dizziness	8	46/2396	27/1917	*P* = 0.28, *I*^2^ = 18%	Fixed-effect model	1.4	0.87, 2.27	0.17
Headache	7	46/2100	48/1625	*P* = 0.63, *I*^2^ = 0	Fixed-effect model	0.83	0.55, 1.24	0.36
Diarrhea	5	18/1855	13/1381	*P* = 0.85, *I*^2^ = 0	Fixed-effect model	1.3	0.65, 2.62	0.46
Nasopharyngitis	7	67/1445	52/1433	*P* = 0.84, *I*^2^ = 0	Fixed-effect model	1.3	0.89, 1.89	0.17
Edema	3	8/559	3/555	*P* = 0.42, *I*^2^ = 0	Fixed-effect model	2.25	0.69, 7.35	0.18
Upper respiratory tract infection	6	44/2303	28/1824	*P* = 0.50, *I*^2^ = 0	Fixed-effect model	1.22	0.75, 1.97	0.42
Cough	6	39/2043	13/1568	*P* = 0.27, *I*^2^ = 22%	Fixed-effect model	2.38	1.27, 4.47	0.007^*∗*^
Arthralgia	4	10/1425	10/953	*P* = 0.40, *I*^2^ = 0	Fixed-effect model	0.93	0.40, 2.18	0.87
Back pain	3	10/428	20/425	*P* = 0.17, *I*^2^ = 44%	Fixed-effect model	0.49	0.23, 1.05	0.07
Influenza	3	16/428	15/425	*P* = 0.53, *I*^2^ = 0	Fixed-effect model	1.07	0.52, 2.19	0.86
Hypotension	2	4/417	7/412	*P* = 0.47, *I*^2^ = 0	Fixed-effect model	0.56	0.16, 1.93	0.36
Dyspepsia	2	8/639	2/636	*P* = 0.98, *I*^2^ = 0	Fixed-effect model	4.05	0.86, 19.19	0.08
Hyperuricemia	2	38/1247	35/776	*P* = 0.39, *I*^2^ = 0	Fixed-effect model	0.71	0.44, 1.15	0.17
Abdominal pain	2	5/417	3/412	*P* = 0.09, *I*^2^ = 66%	Random-effect model	1.19	0.05, 27.98	0.91
